# Public Health Implications of Adapting HIV Pre-exposure Prophylaxis Programs for Virtual Service Delivery in the Context of the COVID-19 Pandemic: Systematic Review

**DOI:** 10.2196/37479

**Published:** 2022-06-07

**Authors:** Pragna Patel, Michael Kerzner, Jason B Reed, Patrick Sean Sullivan, Wafaa M El-Sadr

**Affiliations:** 1 COVID-19 Response Team Centers for Disease Control and Prevention Atlanta, GA United States; 2 Jhpiego Washington, DC United States; 3 Rollins School of Public Health Emory University Atlanta, GA United States; 4 ICAP at Columbia University and Mailman School of Public Health New York, NY United States

**Keywords:** HIV, pre-exposure prophylaxis, COVID-19, virtual service delivery, HIV prevention, public health, systematic review, virtual service, health intervention, digital intervention, health technology, social media platform, telehealth, public health message

## Abstract

**Background:**

The novel coronavirus disease COVID-19 caused by SARS-CoV-2 threatens to disrupt global progress toward HIV epidemic control. Opportunities exist to leverage ongoing public health responses to mitigate the impacts of COVID-19 on HIV services, and novel approaches to care provision might help address both epidemics.

**Objective:**

As the COVID-19 pandemic continues, novel approaches to maintain comprehensive HIV prevention service delivery are needed. The aim of this study was to summarize the related literature to highlight adaptations that could address potential COVID-19–related service interruptions.

**Methods:**

We performed a systematic review and searched six databases, OVID/Medline, Scopus, Cochrane Library, CINAHL, PsycINFO, and Embase, for studies published between January 1, 2010, and October 26, 2021, related to recent technology-based interventions for virtual service delivery. Search terms included “telemedicine,” “telehealth,” “mobile health,” “eHealth,” “mHealth,” “telecommunication,” “social media,” “mobile device,” and “internet,” among others. Of the 6685 abstracts identified, 1259 focused on HIV virtual service delivery, 120 of which were relevant for HIV prevention efforts; 48 pertained to pre-exposure prophylaxis (PrEP) and 19 of these focused on evaluations of interventions for the virtual service delivery of PrEP. Of the 16 systematic reviews identified, three were specific to PrEP. All 35 papers were reviewed for outcomes of efficacy, feasibility, and/or acceptability. Limitations included heterogeneity of the studies’ methodological approaches and outcomes; thus, a meta-analysis was not performed. We considered the evidence-based interventions found in our review and developed a virtual service delivery model for HIV prevention interventions. We also considered how this platform could be leveraged for COVID-19 prevention and care.

**Results:**

We summarize 19 studies of virtual service delivery of PrEP and 16 relevant reviews. Examples of technology-based interventions that were effective, feasible, and/or acceptable for PrEP service delivery include: use of SMS, internet, and smartphone apps such as iText (50% [95% CI 16%-71%] reduction in discontinuation of PrEP) and PrEPmate (OR 2.62, 95% CI 1.24-5.5.4); telehealth and eHealth platforms for virtual visits such as PrEPTECH and IowaTelePrEP; and platforms for training of health care workers such as Extension for Community Healthcare Outcomes (ECHO). We suggest a virtual service delivery model for PrEP that can be leveraged for COVID-19 using the internet and social media for demand creation, community-based self-testing, telehealth platforms for risk assessment and follow-up, applications for support groups and adherence/appointment reminders, and applications for monitoring.

**Conclusions:**

Innovations in the virtual service provision of PrEP occurred before COVID-19 but have new relevance during the COVID-19 pandemic. The innovations we describe might strengthen HIV prevention service delivery during the COVID-19 pandemic and in the long run by engaging traditionally hard-to-reach populations, reducing stigma, and creating a more accessible health care platform. These virtual service delivery platforms can mitigate the impacts of the COVID-19 pandemic on HIV services, which can be leveraged to facilitate COVID-19 pandemic control now and for future responses.

## Introduction

The novel coronavirus disease COVID-19 caused by SARS-CoV-2 threatens to disrupt global progress toward HIV elimination [1]. In response to the COVID-19 pandemic, many countries have employed nonpharmacologic interventions such as lockdowns, social distancing, and restrictions on gatherings to control the spread of SARS-CoV-2. However, other countries with a high burden of COVID-19 have not successfully and universally instituted these mitigation measures at a national level [2]. Countries with limited uptake of mitigation measures are seeing their health care infrastructure overwhelmed with the pandemic due to widespread community transmission, and are thus struggling to provide comprehensive clinical care for COVID-19 and for chronic diseases, including HIV [1,2]. Recent gains in HIV epidemic control may be lost if HIV prevention and treatment services are not maintained. Additionally, the morbidity and mortality of COVID-19 might be increased in the face of uncontrolled chronic diseases and HIV, although there have been conflicting reports among persons living with HIV [3,4].

COVID-19 and HIV both disproportionally affect socially disadvantaged and hard-to-reach populations [4]. Opportunities exist to leverage ongoing public health responses to mitigate the impacts of COVID-19 on HIV services, and novel approaches to care provision might help address both epidemics. For example, the US Ending the HIV Epidemic (EHE) initiative aims to overcome existing social and economic disparities by increasing access to HIV services for vulnerable populations in the United States [5]. In this regard, the aims of EHE to increase services for vulnerable populations align with approaches for controlling the COVID-19 pandemic, which has also exacerbated health inequities [6]. Globally, the public and private sectors have collaborated for years to address the HIV crisis using a public health approach. This has resulted in platforms for service delivery, a health workforce trained in HIV care and treatment, supply chains, and collaboration across a diverse group of stakeholders, including community leaders and governments, to ensure that marginalized populations receive the services they need. Efforts should be made to identify best practices and lessons learned from HIV prevention to lessen the impacts of COVID-19 on HIV programs [7]. The HIV community can sustain progress toward HIV epidemic control by rapidly employing innovations to maintain and extend HIV programming during the COVID-19 pandemic [8]. Additionally, COVID-19–specific education, testing, and vaccination could be integrated into HIV prevention programs, considering that these service delivery platforms are designed to reach vulnerable persons at risk of HIV and the general population.

To ensure that HIV prevention programs are improved to deliver services in the context of limited mobility and strained health systems, we reviewed the literature for adaptations of pre-exposure prophylaxis (PrEP) programs for HIV prevention both prior to and in the time of COVID-19. PrEP is vital to achieving HIV epidemic control and should be prioritized in the context of COVID-19 along with HIV treatment. We describe technological innovations for HIV prevention and PrEP service delivery, and propose a model for virtual PrEP service delivery to ensure HIV prevention interventions reach those most vulnerable during the implementation of COVID-19 mitigation measures.

## Methods

### Literature Search and Review

We performed a review of the literature to identify published peer-reviewed articles about virtual service delivery and related adaptations such as telemedicine (see Multimedia Appendix 1 for the detailed search strategy). PRISMA (Preferred Reporting Items for Systematic Reviews and Meta-Analyses) was used as a guide for this systematic review [9]. We searched the OVID/Medline, Scopus, Cochrane Library, CINAHL, PsycINFO, and Embase databases to identify human studies published between January 1, 2010, and October 26, 2021, to reflect the time period during which innovative technologies for health were introduced. Search terms included “telemedicine,” “telehealth,” “mobile health,” “eHealth,” “mHealth,” “telecommunication,” “social media,” “mobile device,” and “internet,” among others (see Multimedia Appendix 1). The search was limited to articles published in English. We used EndNote X8 (Clarivate Analytics) to compile, clean, categorize, and assess citations. We assessed for risk of bias in a randomized controlled trial (RCT) using the Cochrane risk of bias tool [10].

### Ethics Considerations

This activity was reviewed by the US Centers for Disease Control and Prevention (CDC), and was conducted in compliance with applicable federal law and CDC policy. The activity was determined to meet the requirements of nonresearch and secondary data analysis for a public health response, as defined in 45 CFR 46.102(l). Thus, a protocol was not developed and registered.

### Study Selection

Two authors (PP and MK) screened the titles and abstracts of articles identified from our database search for references to HIV, PrEP, and virtual service delivery using filters in EndNote. Next, the same two authors (PP and MK) reviewed the selected articles’ titles and abstracts to identify those reporting effective adaptations for virtual HIV service delivery, particularly related to PrEP, HIV prevention, and HIV testing, by reporting outcomes related to efficacy, feasibility, and/or acceptability. The full text of articles reporting relevant data and systematic reviews of virtual service delivery interventions were further reviewed. All systematic reviews about innovations of virtual HIV service delivery that focused on adherence and HIV testing were included because both have relevance to PrEP programs.

Studies of interventions were included if they focused on innovations for HIV prevention service delivery, particularly PrEP. We thus included intervention studies that described the use of technology such as apps, use of the internet, SMS text messaging, telemedicine/telehealth, mobile health (mHealth), and eHealth for PrEP. We also included all reviews and meta-analyses of technology innovations pertinent to HIV prevention service delivery, as these data would inform the virtual service delivery model that we aimed to propose. We excluded studies that did not focus on virtual service delivery, focused on prevention of vertical mother-to-child transmission, described protocols, were not in English, or were not accessible (Table 1).

**Table 1:** Inclusion and exclusion criteria.

Parameter	Inclusion criteria	Exclusion criteria
Study topic	Technology innovations for HIV prevention and specifically pre-exposure prophylaxis service delivery, virtual service delivery	Focused on vertical mother-to-child transmission, did not focus on virtual HIV prevention service delivery
Study type	Randomized clinical trials, pre-postevaluations, mixed methods evaluations, surveys, reviews, meta-analyses	Protocols, viewpoints, editorials
Language	English	Language other than English
Time frame	Published after 2010	Published before 2010
Accessible	Able to retrieve publication	Publication was inaccessible

**Figure 1:**
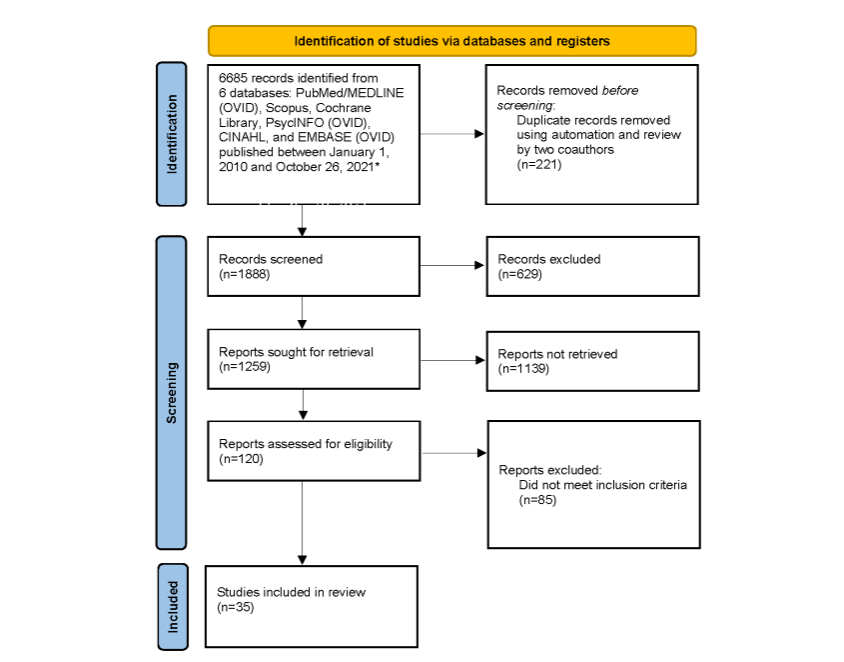
Selection of studies regarding virtual service delivery and HIV. *See Multimedia Appendix 1 for more details.

Figure 1 details the study selection procedure. In addition, the references of papers that were selected were examined to identify other pertinent references. These selections and related data were confirmed by a second reviewer independent of the first reviewer. Studies with missing data were excluded.

### Other Considerations

The RCT was assessed for risk of bias using the Cochrane risk of bias tool [10] and some concerns were identified (Multimedia Appendix 2). Given the limited number of studies and the heterogeneity of studies and their outcomes, it was decided that we could not perform a robust meta-analysis of any given outcome. Thus, related analyses to explore causes of heterogeneity and certainty were not performed.

After reviewing the literature, we considered evidence-based interventions for PrEP service delivery in the time of COVID-19, and developed a virtual service delivery model for implementation to improve HIV prevention services now and in the future. We also suggest how to leverage this model for COVID-19 service delivery to maximize the use of vital health resources. All data and tools used are presented in this manuscript and are publicly available.

## Results

### Characteristics of Included Studies

Of the 6685 abstracts identified, 1888 were specific to virtual service delivery. Of those 1888 articles, 1259 focused on HIV, 120 of which were relevant for HIV prevention efforts; 48 pertained to PrEP and 19 of these focused on evaluations of interventions for virtual service delivery of PrEP; 16 were conducted in the United States and the other three were conducted in Kenya, India, and England [11-29]. One RCT was identified [19]. There were 16 systematic reviews related to virtual HIV care delivery [30-45], 3 of which were related to virtual PrEP delivery [30,42,45]. The remaining articles focused on aspects of care delivery, including telemedicine, use of SMS and the internet, mHealth, and eHealth. (Figure 1). These 16 papers were reviewed and examined for interventions that support virtual care delivery, which were evaluated for efficacy, feasibility, and/or acceptability and could be considered for PrEP service delivery in the time of COVID-19. Three systematic reviews provided pooled estimates [34,39,44].

### Summary of Pertinent Studies

We identified 19 papers [11-29] related to evaluations of interventions for PrEP virtual service delivery. These papers are summarized in Table 2. The primary aim of many of these interventions was to address barriers to PrEP delivery and uptake before the COVID-19 pandemic started in early 2020. For example, the interventions sought to reach people in rural areas or those who were not able to access facilities, as well as eliminating the stigma of PrEP and improving health literacy about many HIV prevention services, including HIV testing, condom use, PrEP, and testing for sexually transmitted infections (STIs). The data presented provide support for the use of social media, smartphone apps, text messaging, and the internet for service delivery and health communication. Two studies described successful PrEP initiation and monitoring using an online platform [17,27], and four studies found that telehealth for PrEP was feasible, acceptable, and effective [13,16,24,25]. Eight studies examined the use of apps [11,18,21,28] and SMS [15,19,22,26] for PrEP service delivery. Of note, only one study, the Enhancing PrEP in Community Settings (EPIC) study, presented results from an RCT [19]. One study described the successful use of the Extension for Community Healthcare Outcomes (ECHO) platform for training of PrEP providers [29]. ECHO, a distance telemonitoring program, has been extensively used to create communities of practice and deliver clinical mentorship to support remote health care provision, which has also been used for PrEP [29,41,46,47].

Of the 16 systematic reviews identified, three were specific to PrEP; one described successful PrEP delivery models, including community-based and home-based approaches [42], and two summarized novel interventions to use technology to improve PrEP availability, adherence, and uptake [41,45]. These include mobile apps that offer PrEP prescribing and preclude an in-person visit, distance mentorship of community PrEP providers, video teleconferencing for provider visits, electronic consults, and using text messaging and mobile/web platforms for PrEP initiation (Table 3) [41,45].

We also summarize systematic reviews that focus on technology innovations to improve HIV testing and adherence in Table 3, because these would also pertain to PrEP programs [30-45]. Most of these reviews focused on interventions to improve adherence to antiretroviral therapy (ART) [31-33,41,43,44]. Two studies reported pooled estimates for improved ART adherence, one for mHealth interventions (pooled odds ratio 2.15, 95% CI 1.18-3.91) and one for eHealth interventions (pooled Cohen *d*=0.25, 95% CI 0.05-0.46). All studies reported positive outcomes; however, the evaluation methods varied and thus have limited comparability.

**Table 2:** Summary of evidence-based interventions for HIV pre-exposure prophylaxis (PrEP) virtual service delivery.

Author, year	Country	Intervention	Sample size	Study period	Study name	Evaluation	Results	Main conclusions
Biello et al [11], 2021	United States	MyChoices app to increase HIV testing and PrEP uptake	11 YMSM^a^	2 months	University of North Carolina/Emory Center for Innovative Technology (iTech)	Baseline and 2-month postbaseline assessments	Mean System Usability Scale (SUS) score was 71 (SD 11.8); 80% (9/11) reported that app was useful	MyChoices app is acceptable to end users
Bond et al [12], 2019	United States	Avatar-led eHealth video	116 African American women aged 16-61 years	6 weeks	PEP^b^ and PrEP for Women	Cross-sectional web-based study with thematic analysis	89% of participants rated the video as good or higher	Utilization of an avatar-led eHealth video fostered education about PEP and PrEP among African American women who have experienced insufficient outreach for biomedical HIV strategies
Chasco et al [13], 2021	United States	Home specimen self-collection kits with central laboratory testing	77 participants offered test kits (35 accepted and 42 refused)	18 months	Iowa TelePrEP	Mixed methods evaluation	Compared to laboratory-site use, kit use was associated with higher completion of extragenital swabs (OR^c^ 6.33, 95% CI 1.20-33.51 for anorectal swabs), but lower completion of blood tests (OR 0.21, 95% CI 0.06-0.73 for creatinine)	Telehealth PrEP programs should offer clients home kits and support clients with blood collection and kit completion
Farley et al [14], 2021	United States	Community engagement and linkage with both virtual and face-to-face models; prepmaryland.org, PrEP telephone/text line, and PrEPme smartphone app	2465 (24 prepmaryland.org; 60 phone line; 168 PrEPme app)	4 years	N/A^d^	Observational programmatic evaluation	Overall success of: prepmaryland.org (4/24, 16.7%), phone line (18/60, 30%), PrEPme app (39/168, 23.2%)	Compared to face-to-face community outreach efforts, all virtual platforms reached lower total numbers, but had greater success in attendance at PrEP visits
Fuchs et al [15], 2018	United States	Mobile health intervention (iText) to support adherence with bidirectional texting	56 MSM^e^	12-week pilot	iPrEx open-label extension study	Pre- and postintervention regression discontinuity analysis	50% reduction in discontinuation of meds (95% CI 16%-71%; *P*=.008)	iText strategy was feasible and acceptable, and improved adherence to PrEP
Hoth et al [16], 2018	United States	Pharmacist-led video visits	186 referrals; 91% MSM	18 months	Iowa TelePrEP	Results at 6 months	Retention was 61%, and 96% completed laboratory tests	Regional telehealth PrEP programs can be developed to offer PrEP widely
Hughes et al [17], 2021	United States	Web-based PrEP service	31 PrEP requesters	5 months	Nurx	Electronic chart review and 90-minute semistructured interviews	Nurx eased barriers to PrEP access through the availability of knowledgeable, willing prescribers, and minimizing embarrassment and discrimination	Nurx produced satisfaction by achieving an acceptable balance between 2 client desires: efficiency and humanity
Liu et al [18], 2019	United States	LYNX app to support HIV testing and PrEP uptake	30 YMSM in focus group and 16 in open pilot	2-month pilot	Adolescent Trials Network iTech U19	SUS and focus groups	Median SUS score=72/100	The LYNX app was feasible and acceptable; well-received, especially the sexual diary and gamification features (sex-positive badges)
Liu et al [19], 2019	United States	Youth-tailored bidirectional text messaging intervention (PrEPmate)	121 participants	36 weeks	EPIC study	Randomized clinical trial (RCT) with some concerns of risk of bias^f^	Participants who received PrEPmate were more likely to attend study visits (OR 2.62, 95% CI 1.24-5.5.4) and have TDF-DP^g^ levels consistent with >4 doses/week (OR 2.05, 95% CI 1.06-3.94)	An interactive text messaging intervention had high acceptability and significantly increased retention and adherence
McLaughlin et al [20], 2016	United States	Tweets about PrEP on Twitter	1435 tweets	4 months	N/A	Poisson regression for propagation rate	Affective tone was a significant predictor of tweet propagation frequency (Wald *χ*^2^_2_=30.997, *P*<.001)	PrEP-related tweets covered a wide range of issues, and affective tone in tweets is a critical factor in predicting propagation
Mitchell et al [21], 2018	United States	Smartphone-based intervention (mSMART)	10 YMSM	4 weeks	mSMART open-label phase 1 trial	Real-time adherence assessment using a camera-based medication event–monitoring tool	Participants reported mean PrEP adherence rates of 91% via daily entries in mSMART	mSMART is feasible and acceptable
Muwonge et al [22], 2018	Kenya	SMS-based surveys to collect data on sexual behaviors and adherence	142 participants from serodiscordant partnerships	24 months	Partners Demonstration Project	Questionnaires	72% preferred SMS surveys to in-person visits	SMS surveys were acceptable and serve as reminders for adherence to PrEP and condom use
Patel et al [23], 2020	India	Peer-delivered, internet-based messaging for HIV testing and condom use	244 participants	12 weeks	CHALO! Pilot	Pre-postsurveys	Increase in HIV testing	Online HIV prevention interventions are feasible and acceptable, and can improve HIV testing rates
Perlson et al [24], 2018	United States	PrEP telenavigation program	139 participants	9 months	“At Distance” PrEP Navigation	Surveys	Increased knowledge of PrEP and linkage to HIV testing and prevention services	PrEP telemedicine can improve PrEP utilization
Refugio et al [25], 2019	United States	Telehealth approach to PrEP initiation	25 YMSM	180 days	PrEPTECH	2 online surveys	At least 75% felt PrEPTECH was confidential, fast, convenient, and easy to use	Telehealth PrEP programs increase access and eliminate barriers such as stigma
Shrestha et al [26], 2020	United States	Text messages over a 4-week intervention period	40 people enrolled in a methadone maintenance program	10 months	Telerivet mobile messaging platform	An audio computer-assisted self-interview (ACASI) was used to assess all quantitative measures and qualitative interviews were semistructured	Mean adherence score of 87.6 (SD 18.6) for having taken PrEP in the past 30 days; mean acceptability (range 0-100) for the daily PrEP reminder was 75.0 (SD 11.7)	Preliminary evidence of the feasibility and acceptability of a text messaging–based approach as a potential tool for primary HIV prevention to improve PrEP adherence and HIV risk reduction among this underserved population
Wang et al [27], 2018	England	Online generic PrEP and therapeutic drug monitoring	293 individuals	6 months	InterPrEP	Testing baseline and every 3-6 months	PrEP drug concentrations were above target; no creatinine elevations were seen; no cases of HIV, hepatitis B or C were noted	Online PrEP services with therapeutic drug monitoring are feasible
Weitzman et al [28], 2021	United States	PrEP adherence mobile app (“Dot”); the Dot intervention combined with personalized pill reminders with positive psychology-based texts	54 culturally diverse YMSM	6 weeks	Dot app	Pre- and posttest evaluation of the impact of the Dot mobile app on self-reported PrEP adherence, PrEP treatment self-efficacy, PrEP knowledge, and intention to practice safe sex	Significant changes in the percentage of participants who reported perfect (100%) PrEP adherence from pre- to posttesting (t_53_=4.458, *P*<.001); PrEP treatment self-efficacy (t_53_=3.067, *P*=.003); and intention to follow safe sex and HIV testing guidelines (t_53_=3.067, *P*=.003).	The Dot app was feasible and effective at improving PrEP adherence for supporting medication adherence among culturally diverse YMSM on PrEP
Wood et al [29], 2018	United States	Project ECHO^h^–PrEP telemonitoring intervention	69 medical providers	2 years	Project ECHO	Pre- and postsurvey	Providers reported that Project ECHO participation helped them stay current on PrEP guidelines, improved knowledge, increased likelihood to prescribe PrEP, and addressed most concerns about prescribing PrEP	It is feasible to incorporate PrEP training into Project ECHO distance telementoring programs as a tool to educate community practitioners and support PrEP prescribing

^a^YMSM: young men who have sex with men.

^b^PEP: postexposure prophylaxis.

^c^OR: odds ratio.

^d^N/A: not applicable.

^e^MSM: men who have sex with men.

^f^Risk of bias was assessed using the Cochrane risk of bias tool [10] (see Multimedia Appendix 2).

^g^TDF-DP: tenofovir diphosphate.

^h^ECHO: Extension for Community Healthcare Outcomes.

**Table 3:** Systematic reviews of technological innovations for improved HIV and pre-exposure prophylaxis (PrEP) service delivery.

Author, year	Innovation(s) examined	Outcomes	Main findings
Catalani et al [30], 2013	62 articles summarizing the use of mobile health (mHealth) technology for HIV/AIDS	N/A^a^	Promising trend toward implementing mHealth innovations that are feasible and acceptable, but they are still in their early stages
Claborn et al [31], 2015	Computer-delivered adherence intervention; 5 randomized controlled trials (RCTs) and 1 single-group pre-posttrial; 5 conducted in the United States and 1 in Canada	Adherence	Computer-delivered adherence interventions are feasible and acceptable among both HIV-positive adolescents and adults
Cooper et al [32], 2017	mHealth interventions, mainly SMS-based. The 41 studies were conducted in 12 countries across North America, South America, Africa, Asia, Europe, and New Zealand	Adherence and health-related behaviors	Significant impacts on a range of outcomes, including adherence, viral load, mental health, and social support
Daher et al [33], 2017	Digital innovations, classified into (1) mHealth-based (SMS/phone calls), (2) internet-based mHealth/eHealth (social media, avatar-guided computer programs, websites, mobile apps, streamed soap opera videos), and (3) combined innovations (including both SMS/phone calls and internet-based mHealth/eHealth). Reviewed 99 studies, 63 (64%) from America/Europe, 36 (36%) from Africa/Asia; 79% (79/99) were clinical trials; 84% (83/99) evaluated impact. Of innovations, 70% (69/99) were mHealth-based, 21% (21/99) were internet-based, and 9% (9/99) were combined. All digital innovations were highly accepted (26/31, 84%) and feasible (20/31, 65%)	Feasibility, acceptability, impact. mHealth-based innovations (SMS) significantly improved antiretroviral therapy (ART) adherence (pooled OR^b^ 2.15, 95% CI 1.18-3.91) and clinic attendance rates (pooled OR 1.76, 95% CI 1.28-2.42); internet-based innovations improved clinic attendance (6/6), ART adherence (4/4), and self-care (1/1), while reducing risk (5/5); combined innovations increased clinic attendance, ART adherence, partner notifications, and self-care	Digital innovations were acceptable, feasible, and generated impact. A trend toward the use of internet-based and combined (internet and mobile) innovations was noted. Large scale-up studies of high quality, with new integrated impact metrics and cost-effectiveness are needed. Findings will appeal to all stakeholders in the HIV/STI global initiatives space
Hightow et al [34], 2015	Synthesis of 66 relevant papers on HIV, technology, and youth	N/A	A growing number of technology-based interventions for HIV prevention and care have been published; however, the majority were published in the United States. Given the disproportionate burden of HIV among adolescents worldwide, there is a need for more broadly expanding eHealth and mHealth to youth globally
Horvath et al [35], 2020	mHealth and other technology-based interventions for HIV testing: 6 efficacy trials and 12 pilot RCTs or quasiexperimental studies; 10 were conducted outside the United States, including countries in sub-Saharan Africa (n=4: Kenya, Tanzania, South Africa), China (n=3), Latin America (n=2: Brazil, Peru), and India (n=1)	Efficacy, feasibility, acceptability	All efficacy trials showed some evidence of efficacy. Most pilot RCTs demonstrated high levels of feasibility and acceptability. Technology-assisted HIV testing interventions may be an important strategy to reach national and global targets for HIV status awareness in the general population and for most at-risk groups
Labelle et al [36], 2020	Summary of 22 papers on use of technology for HIV prevention and PrEP to inform an mHealth app development in Taiwan	N/A	Features identified from studies testing HIV prevention applications for PrEP, such as education and gamification, will be used to formulate features of an HIV prevention app in Taiwan
Maloney et al [37], 2019	eHealth interventions for HIV care and prevention; 113 studies were included with 84 unique interventions. The majority (n=71, 85%) of interventions were developed for users in resource-rich countries. The remaining (n=13, 15%) were intended to address the unique cultural needs of specific communities in low- or middle-income countries	N/A	Robust collection of eHealth interventions in the published literature as well as unpublished interventions still in development. In the published literature, there is an imbalance of interventions favoring education and behavior change over linkage to care, retention in care, and adherence, especially for PrEP
Manby et al [38], 2021	25 RCTs that randomized a total of 15,343 participants: 2356 were randomized to interactive interventions, 5530 to noninteractive interventions, and 5808 to the control condition. Studies were from 10 countries in Africa: 8 in Kenya, 7 in Uganda, and 5 in South Africa; 6 studies reported outcomes related to HIV prevention behaviors	Meta-analyses show that eHealth interventions significantly improved HIV management behaviors (OR 1.21, 95% CI 1.05-1.40; *Z*=2.67; *P*=.008), but not HIV prevention behaviors (OR 1.02, 95% CI 0.78-1.34; *Z*=0.17; *P*=.86). There was no effect for HIV testing or biological outcomes (OR 1.17, 95% CI 0.89-1.54; *Z*=1.10; *P*=.27) compared with minimal intervention control groups	eHealth interventions can improve adherence to ART in sub-Saharan Africa, and serve as important tools to help reduce HIV-related morbidity and mortality as well as HIV transmission
Nelson et al [39], 2020	16 studies: 1 study was a fully powered RCT, 7 were single-arm pilots with pre-postassessments, 4 were pilot RCTs, and 4 tested public health campaigns with postassessments	N/A	All studies found that mHealth approaches were feasible and acceptable; however, most studies were small pilot trials
Schnall et al [40], 2014	13 studies: 5 targeted HIV testing behaviors and 8 focused on decreasing HIV risk behaviors with wed-based education modules, test messaging, chat rooms, social networking	N/A	eHealth has the potential to effectively reduce HIV risk behaviors and increase testing rates. Further evaluations are needed as there was wide variation in interventions and methodological quality
Touger et al [41], 2019	Multiple models of telehealth innovations in the United States (8 studies): *provider to patient* (mobile apps for PrEP prescribing [nurx.com], videoconferencing for PrEP initiation [PrEPTECH, PrEPIOWA, plushcare.com], home-based PrEP [ePrEP]); *provider to provider* (distance learning for community providers [ECHO^c^ Project], electronic consults for PrEP support)	PrEP dissemination and adherence	Technology-based intervention can address gaps in the PrEP care continuum and reach underserved populations; however, costs may impede progress. Platforms to share technology are needed as well as further research to assess scalability and sustainability
Vanhamel et al [42], 2020	Scoping review of PrEP delivery models. The identified service delivery models showed that PrEP services mainly targeted people at high risk of HIV acquisition, with some models targeting specific key populations, mainly men who have sex with men	N/A	PrEP was often delivered centralized and in a clinical or hospital setting; yet, community-based as well as home-based PrEP delivery models were also reported. Providers of PrEP were mainly clinically trained health professionals, but in some rare cases community workers and lay providers also delivered PrEP. In general, in-person visits were used to deliver PrEP. More innovative digital options using mHealth and telemedicine approaches to deliver specific parts of PrEP services are currently being applied in a minority of the service delivery models in mainly high-resource settings. This reflects differentiation of care according to different contextual settings
Velloza et al [43], 2021	Systematic review of adherence support interventions for adolescents. Fifteen oral contraceptive pill (OCP) articles and 26 ART, diabetes, and asthma systematic reviews were included. Interventions that improved medication adherence for OCPs, ART, asthma, and diabetes treatment included reminder text messages, computer-based and phone-based support, and enhanced counseling. Multimonth prescriptions and same-day pill starts also were found to improve OCP adherence and continuation. Adolescent-friendly clinics and peer-based counseling significantly improved ART adherence, and telemedicine interventions improved diabetes medication adherence	Adherence. Enhanced counseling (whether in groups, families, or computer-delivered) and phone-based support (eg, one-way and two-way text messages) improved ART adherence. Peer support interventions and adolescent-friendly services were effective for ART adherence	Interventions that improve medication adherence among youth include enhanced counseling, extended pill supply, adolescent-friendly services, and text message reminders. PrEP programs could incorporate and evaluate such interventions for their impact on PrEP adherence and continuation among at-risk adolescents
Wang et al [44], 2019	eHealth interventions. Twenty-one trials: 8 trials from high-income countries and 13 trials from low- and middle-income countries	Adherence. eHealth interventions significantly improved ART adherence of people living with HIV (pooled Cohen *d*=0.25, 95% CI 0.05-0.46; *P*=.01)	Some of the eHealth interventions may be used as an effective method to increase the ART adherence of people living with HIV
Wong et al [45], 2020	Four studies: one pilot study, three retrospective evaluations (Iowa TelePrEP, PrEPTech, Nurx, PlushCare)	Retention. The percentage of PrEP initiation after the first telehealth appointment ranges from 84% to 94%, and 6-month retention remains relatively high, in the range of 76%-99%	Success could be attributed to the ability of technology to address the barriers of geographic distance and social stigma faced by those who would otherwise have limited access to care. The use of telemedicine for PrEP is generally viewed by users as easy, fast, and convenient

^a^N/A: not applicable.

^b^OR: odds ratio.

^c^ECHO: Extension for Community Healthcare Outcomes.

### PrEP Virtual Service Delivery Model

**Figure 2:**
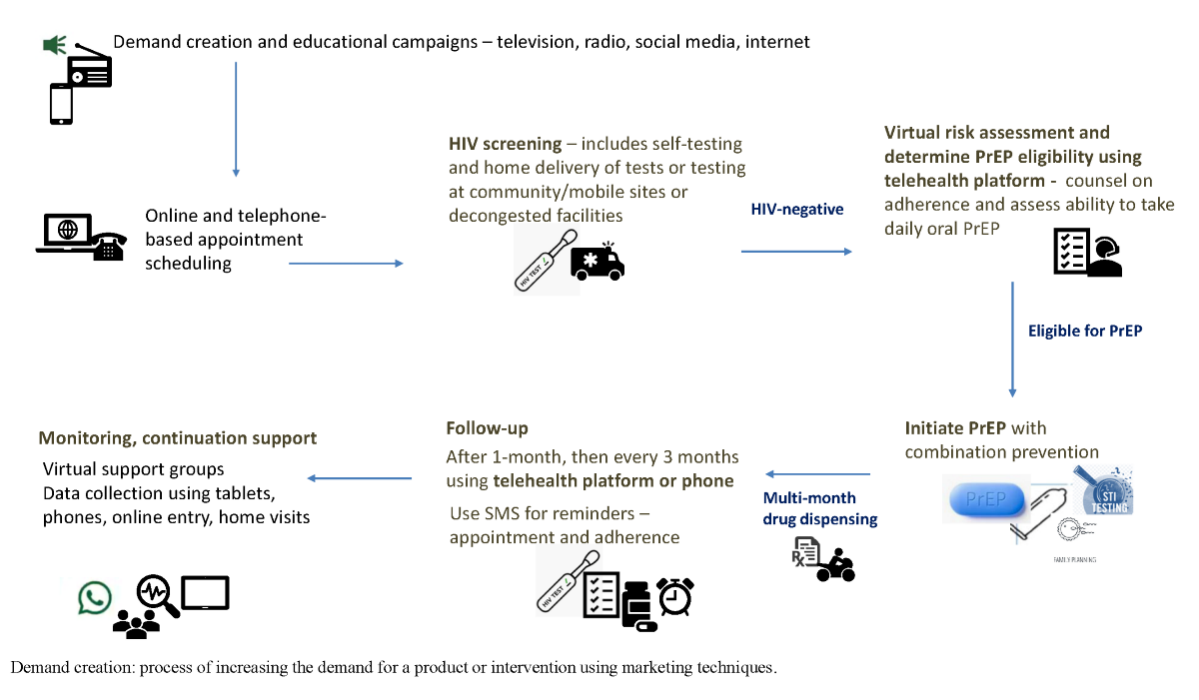
Model of differentiated virtual HIV pre-exposure prophylaxis (PrEP) delivery.

According to the evidence-based innovations identified from the literature review and those implemented during the COVID-19 pandemic, we suggest a comprehensive model for virtual PrEP service delivery (Figure 2) that includes a combination of interventions such as internet for demand creation and risk assessment, telehealth platforms for visits and training, multimonth dispensing and medication delivery, community-based and self-testing for HIV, and smartphone apps for follow-up reminders and adherence support groups [11-45,48-51]. Regarding monitoring and follow-up, quarterly PrEP monitoring is acceptable and preferred with in-person follow-up but also with telehealth [52].

### Leveraging Adaptations for COVID-19 Service Delivery

Although our primary goal was to consider how innovative PrEP program delivery methods could be used to mitigate the impact of COVID-19 on PrEP services, it is also clear that these PrEP delivery methods have the potential for COVID-19 prevention and control (Table 4). Examples include: (1) demand creation using traditional (radio, television) and social media platforms for HIV prevention and COVID-19 messaging; (2) service delivery with decentralized care, moving from facility to community, including home-based and mobile delivery (for HIV and/or COVID-19 testing and multimonth PrEP prescription refills); virtual platforms for follow-up appointments (telehealth), such as risk assessment, lab assessment, and adherence counseling for PrEP; mental health counseling; using apps (eg, WhatsApp) for support groups and mobile device SMS for adherence reminders; (3) training and education with the use of web-based platforms for education and training of health care workers about HIV prevention (and developments in COVID-19 prevention and management) to promote task-shifting (eg, ECHO project); (4) monitoring and evaluation, involving web-based and app-based data entry using tablets and/or mobile devices of PrEP core indicators; and (5) supply chain vulnerabilities, involving working with governments to enact policies that allow for continued manufacturing of commodities and novel procurement and distribution platforms (electronic prescriptions, mail order, direct drug delivery).

**Table 4:** Leveraging adaptations to HIV pre-exposure prophylaxis (PrEP) service delivery in the time of COVID-19.

PrEP program component	Adaptation	Evidence/reference	Contribution to COVID-19 response
Demand creation	Add web-based and social media platforms (TikTok videos, ads on Facebook and Instagram, pop-ups in apps like Tinder); continue campaigns and traditional methods (TV and radio)	[12,18-20,23,28]	Incorporate COVID-19 messaging, including information about social distancing and face masks, into the PrEP messaging and HIV education materials and campaigns
Service delivery	Decentralize care to decongest the clinics/facilities; virtual platforms for scheduling and appointments with maps for locations of mobile sites; use community and mobile delivery (eg, tests and medications); virtual risk assessment (using internet and/or apps); scale-up telehealth for consultation with PrEP provider for review of risk assessment and test results, adherence counseling for PrEP, and mental health counseling in general; support groups (eg, WhatsApp) and mobile device SMS for adherence and appointment reminders	[11-45,48,52]	Provide COVID-19 services as prevention and treatment modalities become available. This platform could be used to deliver the COVID-19 vaccine
Drug delivery	Multimonth prescriptions for PrEP, home delivery using postal service, mobile pharmacies	[13,16,24,25,27,49-51]	Delivery of pharmacologic interventions for COVID-19
Testing	Home-based testing and self-testing, mobile testing sites (eg, drive-through sites), home delivery (postal service, health care workers), community delivery (eg, at pharmacies, faith-based centers, vending machines)	[13,16,17,25,35,41,45]	Delivery of COVID-19 testing
Training and education	Web-based platforms for education and training of health care workers about HIV prevention, particularly PrEP (eg, ECHO^a^ Project)	[29,41,46,47]	Include developments in COVID-19 management to promote task-shifting so PrEP providers are knowledgeable about COVID-19 diagnostics, treatments, and prevention interventions
Monitoring and evaluation	Web-based and app-based data entry using tablets and/or mobile devices of PrEP core indicators and syndromic surveillance for acute HIV infection; system to monitor the PrEP cascade (number who tested HIV-negative, number eligible/offered PrEP, number who initiated PrEP, number adherent and retained)	[52]	Syndromic surveillance for COVID-19 symptoms and data collection of indicators related to COVID-19 response: testing uptake and results, contact-tracing outcomes, severity of illness, uptake of services, vaccine recipients, adverse events related to vaccines
Supply chain	Working with governments to enact policies and agreements that allow for continued manufacturing of commodities, and novel procurement and distribution platforms (electronic prescriptions, mail order, direct drug delivery)	N/A^b^	Leverage to include COVID-19 commodities such as vaccines
Community engagement	Engage community leaders in education of PrEP, including benefits; institute virtual peer-to-peer support groups; advocacy for PrEP to protect persons vulnerable to HIV acquisition	[14,20,22,23]	Education about COVID-19 prevention, treatment, and control, as well as advocacy for services needed by disenfranchised persons

^a^ECHO: Extension for Community Healthcare Outcomes.

^b^N/A: not applicable.

## Discussion

### Principal Findings

COVID-19 mitigation measures such as physical distancing and lockdowns have created significant challenges for HIV and PrEP programming [8]. This systematic review is unique in that it provides a comprehensive overview of specific technology-based interventions as well as differentiated service delivery models that may be critical to program adaptation during COVID-19. Our findings demonstrate that interventions developed before COVID-19, dating back to 2013, for successful adaptation of PrEP programs for virtual service delivery for HIV testing, ART adherence, and PrEP exist and are currently in use. Innovations such as telemedicine; using the internet and smartphone apps for demand creation, support groups, and follow-up reminders; and multimonth dispensing with mobile pharmacies are evidence-based interventions designed to address distance to services and improve convenience. These innovations might also be particularly impactful in the context of COVID-19 [11-29]. Our review also identified examples of models for virtual service delivery that use technology to support PrEP users, such as PrEPTECH, IowaTelePrEP, and telehealth-led PrEP service delivery [41,42,45]. However, these models address some but not all aspects of PrEP implementation, such as commodity procurement and the ability to purchase medications, which is challenging when countries are locked down and companies cannot supply and distribute drugs.

To build upon the current literature and suggest a practical application for innovative technological adaptations, we used findings from the literature review to develop an example of a model of virtual PrEP delivery, which incorporates innovations identified in our literature search. We identified evidence-based interventions that could adapt the current PrEP service delivery platform to provide decentralized, virtual care. This model would allow for continued PrEP service delivery in the face of COVID-19 mitigation strategies but also may improve our ability to engage hard-to-reach populations who do not access care at facilities. The model is also aligned with approaches already described in some countries. For example, in Brazil, at the initial teleconsultation, individuals are assessed for PrEP by phone and undergo HIV rapid testing. Individuals receive a digital prescription to retrieve a 120-day PrEP supply plus two HIV self-test kits, because home delivery was deemed unaffordable. Subsequent follow-up teleconsultations are performed remotely by phone call, including instructions for the HIV self-test performance and the results are shared by digital photo. This approach was successful in maintaining PrEP services, including uptake, as part of the Implementation PrEP Study (ImPrEP) project [48]. In addition, community pharmacists can deliver drugs and manage minor ailments, which supports the use of task-shifting [49]. Our model can be implemented in countries with widespread access to the internet and smartphones. However, implementation could be challenging in areas where such technologies are limited or unreliable.

Globally, differentiated service delivery models to improve the reach of PrEP and HIV programs, to decongest facilities, and to limit exposure to SARS-CoV-2 are recommended [53]. Our model of service delivery could help to maintain PrEP services in resource-poor settings in all countries, including the United States and Canada, and might improve the program’s ability to reach those most vulnerable by improving access to services and eliminating stigma associated with accessing facilities known to provide HIV services. Sexual and reproductive services could be leveraged to offer virtual HIV prevention services, particularly PrEP, as STI testing, condoms, and contraception should be offered with PrEP. Program evaluations are needed to understand the broader feasibility and impact of virtual service delivery models in low- and middle-income countries. To ensure that persons at substantial risk of HIV continue to benefit from PrEP, approaches to scale up virtual service delivery are underway in many countries [54]. HIV prevention services could also be leveraged for related prevention interventions such as STI testing, and to enhance the COVID-19 pandemic response.

Health care workers providing PrEP services can be trained virtually to deliver COVID-19 services, including education about mitigation measures and vaccination using online platforms [41,45]. The internet and smartphone apps can be used for service delivery such as intake assessments and appointment reminders or other public health communications such as contact-tracing programs alerting someone of exposure to SARS-CoV-2. COVID-19 testing can be offered through HIV testing platforms in the facility and community to create efficiencies. In addition, other prevention modalities for both HIV and COVID-19 could be delivered through the HIV prevention platform by leveraging the virtual service delivery of PrEP for COVID-19. For example, once COVID-19 vaccines [55] become widely available, PrEP service delivery could be leveraged for safe, widespread delivery by offering vaccination to clients who present for HIV testing.

These adaptations should be instituted with engagement of governments, stakeholders, and community leaders. Community engagement is fundamental to the success of syndemic control; community leaders can be influential and are key for disseminating factual information. Efforts should be made to accurately forecast needs, in terms of funding, personnel, commodities, and others, and to allocate resources such that resources are not exhausted and are adequately redistributed as needed. Systems should be agile and adopt new advances in HIV prevention rapidly. Although our review was motivated by concern regarding service interruptions related to COVID-19, programs should be developed both for mitigating current service interruptions and for increasing efficiencies and creating more resilience to future causes of service interruptions. A recent study of HIV service disruption in sub-Saharan Africa highlighted that the most important priority to avoid additional deaths due to HIV during the COVID-19 pandemic was to maintain the supply of antiretroviral drugs for people living with HIV [56]. Provision of other HIV prevention interventions to prevent an increase in HIV incidence was also deemed necessary [56]. Therefore, our model of virtual service delivery might be relevant for maintaining and achieving low levels of HIV incidence.

### Limitations

One important challenge that has not been addressed through our review and our model is the maintenance of supply chain and procurement mechanisms to ensure that HIV commodities such as drugs and tests remain available. National-level lockdowns have negatively impacted major pharmaceutical manufacturers, along with the global supply chain of drugs and medical commodities. Governments must ensure that HIV commodities procurement and delivery are maintained as essential services during pandemics that require lockdowns and quarantine for control. Governments should enact policies that allow for continued manufacturing of commodities and novel procurement and distribution platforms. Our literature review had other important limitations. We focused on HIV programs and may have missed relevant innovations used for other types of service delivery. We were not able to conduct meta-analyses for each PrEP intervention (Table 2) identified in our search given the small number of papers and the heterogeneity between studies, particularly of methodology. This limited our ability to conduct analyses related to syntheses of outcomes data. Lastly, as our search focused on service delivery, the review did not yield papers about policy needs related to virtual service delivery, which was outside the scope of our primary objectives.

### Conclusions

Although vaccines are critical to effectively controlling the COVID-19 pandemic, there are ongoing threats to COVID-19 control (and therefore to sustaining HIV prevention and care programs); most notably, the identification of variant strains with increased transmissibility and immune escape from current vaccines poses a significant threat to infection control [57]. COVID-19 control measures may need to continue to limit the spread of SARS-CoV-2 infection due to these variants in some countries [57]. The COVID-19 pandemic has catalyzed a new reality of virtual care [58]. Virtual health service delivery could improve accessibility and affordability of health care, and might improve health inequities, especially for people who are not proximate to care facilities, during COVID-19. However, this requires further investigation. There are also other relevant and newer technologies that have not yet been studied in this context. For example, machine learning can be used to identify individuals who might benefit from HIV testing, PrEP, and other risk reduction strategies [59]. Wearable devices with biosensing capabilities could be updated to improve adherence to daily medications; to provide location information for testing and pharmacies services and/or facilitate contact tracing; and to provide notifications to maintain social distancing [60]. Further investigation is warranted to assess the feasibility, acceptability, and effectiveness of these new technologies and understand their role in public health and medicine.

Innovations in virtual service provision of PrEP occurred before COVID-19 but have new relevance in the COVID-19 pandemic. The United Nations Program on HIV and AIDS (UNAIDS) 2020 target of 3 million on PrEP was not achieved; without innovations and evolution of standard models of delivery, reaching the 2025 target of 95% of those at risk using effective combination prevention options may be similarly beyond reach. Substantial gains in HIV care and the intended acceleration toward global HIV epidemic control may be lost. The innovations we describe might strengthen HIV prevention service delivery in the long run by engaging traditionally hard-to-reach or remote populations, reducing stigma, and by also creating a more accessible health care platform. These are platforms that can be leveraged both to mitigate the impacts of the COVID-19 pandemic on HIV services, and to support interventions for the COVID-19 response and facilitate pandemic control directly now and in the future.

## Appendix

Multimedia Appendix 1 Literature review search strategy.

Multimedia Appendix 2 Risk of bias arising from the randomization process for the randomized control trial [19] using the Risk of Bias 2 tool.

## Abbreviations

ART: antiretroviral therapy
CDC: Centers for Disease Control and Prevention
ECHO: Extension for Community Healthcare Outcomes
EHE: Ending the HIV Epidemic
EPIC: Enhancing PrEP in Community Settings
ImPrEP: Implementation PrEP Study
mHealth: mobile health
PrEP: pre-exposure prophylaxis
PRISMA: Preferred Reporting Items for Systematic Reviews and Meta-analyses
RCT: randomized controlled trial
STI: sexually transmitted infection
UNAIDS: United Nations Program on HIV and AIDS
