# Supplementary material for: Public Health Implications of Adapting HIV Pre-exposure Prophylaxis Programs for Virtual Service Delivery in the Context of the COVID-19 Pandemic: Systematic Review
Source: JMIR Public Health Surveill. 2022 Jun 7;8(6):e37479. doi: 10.2196/37479 (PMC9177169; doi:10.2196/37479)
Supplement: Multimedia Appendix 1 [file publichealth_v8i6e37479_app1.docx]

**Appendix 1**. Literature review search strategy

| **Database** | **Strategy** |
| --- | --- |
| **Medline**  **(OVID)**  **1946-** | *Telemedicine/ OR (telemedicine OR tele-medicine OR telehealth OR mobile health OR ehealth OR mhealth OR ((healthcare OR health care OR health service* OR clinical care OR (access* ADJ2 care) OR health education OR health information OR health literacy OR pandemic* OR outbreak* OR syndromic surveillance) ADJ5 (telecommunication* OR social media OR mobile phone* OR mobile device* OR smartphone* OR smart phone* OR cell phone* OR internet OR web-based OR app OR apps OR text messag* OR Facebook OR Twitter OR Instagram OR reddit OR whatsapp OR tiktok))).ti,ab,kf.  AND  COVID-19* OR coronavirus OR corona virus OR HIV* OR human immunodeficiency virus* OR (review OR metaanalysis OR meta-analysis).ti  Limit 2010 - ; Abstract Available |
| **Embase**  **(OVID)**  **1988-** | *Telemedicine/ OR (telemedicine OR tele-medicine OR telehealth OR mobile health OR ehealth OR mhealth OR ((healthcare OR health care OR health service* OR clinical care OR (access* ADJ2 care) OR health education OR health information OR health literacy OR pandemic* OR outbreak* OR syndromic surveillance) ADJ5 (telecommunication* OR social media OR mobile phone* OR mobile device* OR smartphone* OR smart phone* OR cell phone* OR internet OR web-based OR app OR apps OR text messag* OR Facebook OR Twitter OR Instagram OR reddit OR whatsapp OR tiktok))).ti,ab,kw.  AND  COVID-19* OR coronavirus OR corona virus OR HIV* OR human immunodeficiency virus* OR (review OR metaanalysis OR meta-analysis).ti  NOT pubmed/medline  Limit 2010 -; Abstract Available |
| **PsycInfo**  **(OVID)**  **1987-** | *Telemedicine/ OR (telemedicine OR tele-medicine OR telehealth OR mobile health OR ehealth OR mhealth OR ((healthcare OR health care OR health service* OR clinical care OR (access* ADJ2 care) OR health education OR health information OR health literacy OR pandemic* OR outbreak* OR syndromic surveillance) ADJ5 (telecommunication* OR social media OR mobile phone* OR mobile device* OR smartphone* OR smart phone* OR cell phone* OR internet OR web-based OR app OR apps OR text messag* OR Facebook OR Twitter OR Instagram OR reddit OR whatsapp OR tiktok))).ti,ab,sh.  AND  COVID-19* OR coronavirus OR corona virus OR HIV* OR human immunodeficiency virus* OR (review OR metaanalysis OR meta-analysis).ti  Limit 2010 -; Abstract Available |
| **CINAHL**  **(EbscoHost)** | (MM "Telemedicine") OR (MM "Telehealth") OR (telemedicine OR tele-medicine OR telehealth OR “mobile health” OR ehealth OR mhealth OR ((healthcare OR “health care” OR “health service*” OR “clinical care” OR (access* N2 care) OR “health education” OR “health information” OR “health literacy” OR pandemic* OR outbreak* OR “syndromic surveillance”) N5 (telecommunication* OR “social media” OR “mobile phone”* OR “mobile device*” OR smartphone* OR “smart phone*” OR “cell phone*” OR internet OR web-based OR app OR apps OR “text messag*” OR Facebook OR Twitter OR Instagram OR reddit OR whatsapp OR tiktok)))  AND  COVID-19* OR coronavirus OR “corona virus” OR HIV* OR “human immunodeficiency virus*” OR (TI (review OR metaanalysis OR meta-analysis))  Limit 2010 - ; Abstract Available |
| **Cochrane Library** | [mh telemedicine] OR (telemedicine OR tele-medicine OR telehealth OR “mobile health” OR ehealth OR mhealth OR ((healthcare OR “health care” OR “health service*” OR “clinical care” OR (access* N2 care) OR “health education” OR “health information” OR “health literacy” OR pandemic* OR outbreak* OR “syndromic surveillance”) N5 (telecommunication* OR “social media” OR “mobile phone”* OR “mobile device*” OR smartphone* OR “smart phone*” OR “cell phone*” OR internet OR web-based OR app OR apps OR “text messag*” OR Facebook OR Twitter OR Instagram OR reddit OR whatsapp OR tiktok))):ti,ab  AND  (COVID-19* OR coronavirus OR “corona virus” OR HIV* OR “human immunodeficiency virus*”):ti,ab  Limit 2010 - ; Abstract Available |
| **Scopus** | TITLE-ABS-KEY(telemedicine OR tele-medicine OR telehealth OR “mobile health” OR ehealth OR mhealth OR ((healthcare OR “health care” OR “health service*” OR “clinical care” OR “access to care” OR “health education” OR “health information” OR “health literacy” OR pandemic* OR outbreak* OR “syndromic surveillance”) W/5 (telecommunication* OR “social media” OR “mobile phone”* OR “mobile device*” OR smartphone* OR “smart phone*” OR “cell phone*” OR internet OR web-based OR app OR apps OR “text messag*” OR Facebook OR Twitter OR Instagram OR reddit OR whatsapp OR tiktok))) AND (TITLE-ABS-KEY(COVID-19* OR coronavirus OR “corona virus” OR HIV* OR “human immunodeficiency virus*”) OR TITLE(review OR metaanalysis OR meta-analysis)) AND PUBYEAR > 2009 AND NOT INDEX(medline)  Limit 2010 - ; Abstract Available |
