# Supplementary material for: Public Health Implications of Adapting HIV Pre-exposure Prophylaxis Programs for Virtual Service Delivery in the Context of the COVID-19 Pandemic: Systematic Review
Source: JMIR Public Health Surveill. 2022 Jun 7;8(6):e37479. doi: 10.2196/37479 (PMC9177169; doi:10.2196/37479)
Supplement: Multimedia Appendix 2 [file publichealth_v8i6e37479_app2.docx]

### **Appendix 2.** The Risk of Bias 2 tool (part 2): Risk of bias arising from the randomization process for reference 10

| **Signaling questions** | **Elaboration** | **Response options** |
| --- | --- | --- |
| **1.1 Was the allocation sequence random?**  **Y** | Answer ‘Yes’ if a random component was used in the sequence generation process. Examples include computer-generated random numbers; reference to a random number table; coin tossing; shuffling cards or envelopes; throwing dice; or drawing lots. Minimization is generally implemented with a random element (at least when the scores are equal), so an allocation sequence that is generated using minimization should generally be considered to be random.  Answer ‘No’ if no random element was used in generating the allocation sequence or the sequence is predictable. Examples include alternation; methods based on dates (of birth or admission); patient record numbers; allocation decisions made by clinicians or participants; allocation based on the availability of the intervention; or any other systematic or haphazard method.  Answer ‘No information’ if the only information about randomization methods is a statement that the study is randomized.  In some situations a judgement may be made to answer ‘Probably no’ or ‘Probably yes’. For example, , in the context of a large trial run by an experienced clinical trials unit, absence of specific information about generation of the randomization sequence, in a paper published in a journal with rigorously enforced word count limits, is likely to result in a response of ‘Probably yes’ rather than ‘No information’. Alternatively, if other (contemporary) trials by the same investigator team have clearly used non-random sequences, it might be reasonable to assume that the current study was done using similar methods. | Y/PY/PN/N/NI |
| **1.2 Was the allocation sequence concealed until participants were enrolled and assigned to interventions?**  **NI** | Answer ‘Yes’ if the trial used any form of remote or centrally administered method to allocate interventions to participants, where the process of allocation is controlled by an external unit or organization, independent of the enrolment personnel (e.g. independent central pharmacy, telephone or internet-based randomization service providers).  Answer ‘Yes’ if envelopes or drug containers were used appropriately. Envelopes should be opaque, sequentially numbered, sealed with a tamper-proof seal and opened only after the envelope has been irreversibly assigned to the participant. Drug containers should be sequentially numbered and of identical appearance, and dispensed or administered only after they have been irreversibly assigned to the participant. This level of detail is rarely provided in reports, and a judgement may be required to justify an answer of ‘Probably yes’ or ‘Probably no’.  Answer ‘No’ if there is reason to suspect that the enrolling investigator or the participant had knowledge of the forthcoming allocation. | Y/PY/PN/N/NI |
| **1.3 Did baseline differences between intervention groups suggest a problem with the randomization process?**  N | *Note that differences that are compatible with chance do not lead to a risk of bias. A small number of differences identified as ‘statistically significant’ at the conventional 0.05 threshold should usually be considered to be compatible with chance.*  Answer ‘No’ if no imbalances are apparent or if any observed imbalances are compatible with chance. Answer ‘Yes’ if there are imbalances that indicate problems with the randomization process, including:   1. substantial differences between intervention group sizes, compared with the intended allocation ratio; or 2. a substantial excess in statistically significant differences in baseline characteristics between intervention groups, beyond that expected by chance; or | Y/PY/PN/N/NI |

|  | 1. imbalance in one or more key prognostic factors, or baseline measures of outcome variables, that is very unlikely to be due to chance and for which the between-group difference is big enough to result in bias in the intervention effect estimate.   Also answer ‘Yes’ if there are other reasons to suspect that the randomization process was problematic:   1. excessive similarity in baseline characteristics that is not compatible with chance.   Answer ‘No information’ when there is no *useful* baseline information available (e.g. abstracts, or studies that reported only baseline characteristics of participants in the final analysis).  The answer to this question should not influence answers to questions 1.1 or 1.2. For example, if the trial has large baseline imbalances, but authors report adequate randomization methods, questions 1.1 and 1.2 should still be answered on the basis of the reported adequate methods, and any concerns about the imbalance should be raised in the answer to the question 1.3 and reflected in the domain-level risk-of-bias judgement.  Trialists may undertake analyses that attempt to deal with flawed randomization by controlling for imbalances in prognostic factors at baseline. To remove the risk of bias caused by problems in the randomization process, it would be necessary to know, and measure, all the prognostic factors that were imbalanced at baseline. It is unlikely that all important prognostic factors are known and measured, so such analyses will at best reduce the risk of bias. If review authors wish to assess the risk of bias in a trial that controlled for baseline imbalances in order to mitigate failures of randomization, the study should be assessed using the ROBINS-I tool. |  |
| --- | --- | --- |
| **Risk-of-bias judgement**  **Some concerns** | See [Table 3,](#_bookmark27) [Table 4](#_bookmark28) and [Figure 1.](#_bookmark29) | Low / High / Some concerns |
| Optional: What is the predicted direction of bias arising from the randomization process? | If the likely direction of bias can be predicted, it is helpful to state this. The direction might be characterized either as being towards (or away from) the null, or as being in favor of one of the interventions. | NA / Favors experimental / Favors comparator / Towards null /Away from null / Unpredictable |
